# Improved Formula for the Stress Intensity Factor of Semi-Elliptical Surface Cracks in Welded Joints under Bending Stress

**DOI:** 10.3390/ma10020166

**Published:** 2017-02-13

**Authors:** Yang Peng, Chao Wu, Yifu Zheng, Jun Dong

**Affiliations:** 1 College of Civil Engineering, Nanjing Tech University, Nanjing 211816, China; yang.peng@njtech.edu.cn (Y.P.); zhengyifu-nj@bewg.net.cn (Y.Z.); 2 School of Transportation Science and Engineering, Beihang University, Beijing 100191, China; wuchao@buaa.edu.cn; 3 Nanjing Municipal Design and Research Institute Co. Ltd., Nanjing 211008, China

**Keywords:** stress intensity factor, welded joints, bending stress, fracture mechanics

## Abstract

Welded joints are prone to fatigue cracking with the existence of welding defects and bending stress. Fracture mechanics is a useful approach in which the fatigue life of the welded joint can be predicted. The key challenge of such predictions using fracture mechanics is how to accurately calculate the stress intensity factor (SIF). An empirical formula for calculating the SIF of welded joints under bending stress was developed by Baik, Yamada and Ishikawa based on the hybrid method. However, when calculating the SIF of a semi-elliptical crack, this study found that the accuracy of the Baik-Yamada formula was poor when comparing the benchmark results, experimental data and numerical results. The reasons for the reduced accuracy of the Baik-Yamada formula were identified and discussed in this paper. Furthermore, a new correction factor was developed and added to the Baik-Yamada formula by using theoretical analysis and numerical regression. Finally, the predictions using the modified Baik-Yamada formula were compared with the benchmark results, experimental data and numerical results. It was found that the accuracy of the modified Baik-Yamada formula was greatly improved. Therefore, it is proposed that this modified formula is used to conveniently and accurately calculate the SIF of semi-elliptical cracks in welded joints under bending stress.

## 1. Introduction

Welded structures are constructed by welding various steel plates together. Weld defects are occasionally found in the welded joints and can either be introduced during the fabrication process or generated in service. Welded joints are highly sensitive to welding defects, which may become crack initiation sites. For instance, in steel bridges, the orthotropic steel decks and intersecting attachments of the box-girders always suffer from fatigue cracking due to the cyclic bending stress introduced by the vehicle wheel loading [1,2]. Therefore, the ability to evaluate the fatigue behavior of a welded joint under bending stress is of crucial significance.

The fracture mechanics approach is a useful method in which the fatigue behavior of welded joints can be evaluated. Normally, the Paris law is used to calculate the fatigue crack growth rate, the accuracy of which is largely dependent on the determination of the SIF. However, it is always difficult to obtain the SIF of various typical cracks like semi-elliptical surface cracks of welded joints, due to their complex geometry and high stress concentrations. During the last decade, theoretical, numerical and empirical approaches have been primarily focused on the determination of the SIF for different surface crack shapes, loading types and boundary conditions [3,4]. Recently, the SIF has been extended to welded joints in the form of notch stress intensity factor (NSIF) concept developed by Lazzarin et al. [5,6,7]. The NSIF controls the crack initial life of the welded joints [8], and the SIF is normally used to determine the crack propagation life. Three common approaches: the weight function method; the numerical method; and the hybrid method, have been used to calculate the SIF for the crack propagation simulation of welded joints.

The weight function method was used to determine the SIF of the welded joints through two steps [9,10]. The first step was to determine the reference SIF solution for certain welded joints as the weight function solution. The second step was to obtain the stress field at the position of the fatigue crack in an uncracked body. The stress distribution of the uncracked body is usually calculated by using the finite element method (FEM). When the weight function and the stress field are obtained, they can be used to determine the SIF for any arbitrary loading on the crack faces. Recently, the weight function method has been extended to welded tubular joints [11] and laser-welded joints [12]. However, the existing weight functions are only applicable for one-dimensional stress distribution, and stress distribution can be two-dimensional in welded joints. Recently, Lindroth et al. [13] suggested a weight function for semi-elliptical surface cracks in T-shaped welded joints. This weight function can be used to determine the SIF in situations with two-dimensional stress distribution. Another important issue is to find a simple method to describe the stress field of the welded joints. Two-dimensional stress distribution cannot be easily represented by a polynomial. Goyal et al. [14] proposed a robust stress analysis method for T-shaped welded joints, which improved the feasibility of the weight function method.

Numerical methods have been developed to determine the SIF of welded joints, such as the finite element method (FEM) [15], the extended finite element method (XFEM) [16,17], and the boundary element method (BEM) [18,19]. The accuracy of the computed SIF is dependent on many factors, including the type of elements; mesh quality; mesh refinement; integration schemes; and the shape of the welding around the crack front. These factors control the accuracy of the stress and displacement fields obtained from the numerical models of the welded joints. Branco et al. [20] proposed some guidelines for the computed SIF by the FEM. Mukhopadhyay et al. [21] provided some suggestions when computing the SIF by the BEM. Msekh et al. [22] explained the advantages and disadvantages of the XFEM. However, these numerical methods often rely on complex mesh design and element selection, making them time consuming and always require experimental validation.

The hybrid method is an empirical method [23], which has attracted increasing attention due to its desirable accuracy and rapid calculation of the SIF. Currently, Japanese Society of Steel Construction (JSSC) [24], British Standards Institution (BSI) [25] and the International Institute of Welding (IIW) fatigue document [26] are the three major fatigue design specifications to provide the SIF formula for the calculation of the SIF for welded joints and are all derived from the hybrid method. In BSI [25] and the IIW fatigue document [26], the SIF of a surface crack in welded joints under tension or bending stress is predicted by multiplying the weld toe magnification factor (*M*_K_ factor) [27] with the SIF of a semi-elliptical surface crack on a rectangular plate [28] (referred to as the Raju-Newman solution in the following discussion). The *M*_K_ factor reflects the local stress concentration due to the welding [29] and is regressed from the data of FEM [30]. This method has proved to be extremely accurate for the cracking along the weld toe. However, for other cracks in welded joints, the Raju-Newman solution was unsuitable for describing their fatigue behaviors [31,32] as it cannot be used to describe the SIF of a surface crack at the weld root and furthermore, the physical meanings of several correction factors in the Raju-Newman solution have not been clarified [33]. Comparatively, the physical meanings of the correction factors in the SIF expression in JSSC [24] are always legible. JSSC is applicable for the SIF of cracks at both welded root and toe positions. For example, the geometry correction factor (*F*_G_ factor) in JSSC reflects the local stress concentration due to the welding, and is directly calculated by the FEM [34]. However, the SIF formula in JSSC is only suitable for cracks under tensile stress, and an SIF formula for bending stress is absent. In 2011, Baik et al. [35] proposed an SIF formula for welded joints under bending stress (called the Baik-Yamada formula in the following discussion). This formula can be used for the rapid and convenient determination of the SIF under bending stress [35]; however, Xiao et al. [18] illustrated that the S-N curves obtained by the Baik-Yamada formula were less accurate than the results obtained by the Raju-Newman formula.

This paper also found that that the accuracy of the Baik-Yamada formula was not satisfactory, and the essential reasons were identified and are discussed in detail. Subsequently, the correction factors in the Baik-Yamada formula were modified, based on the analytical SIF solution. The numerical solutions of the modified Baik-Yamada formula (called the modified formula in the following discussions) were developed through the regression of the finite element analysis. Finally, the modified formula was validated using the existing experimental and numerical data available in the literature.

## 2. Accuracy of the Baik-Yamada Formula

The hybrid method is basically a weight function method [23]. Various factors that have different influences on the SIF are isolated and then multiplied to estimate the SIF of welded joints, expressed as Equation (1) [23].

(1) $$K=F_{s}F_{T}F_{E}F_{W}F_{G}\sigma \sqrt{\pi a}$$

where σ is the applied stress; *a* is the crack depth; *F_s_* is a free surface correction factor; *F_T_* is a finite thickness factor; *F_W_* is a finite width factor; *F_E_* is a crack shape correction factor; and *F_G_* is a geometry correction factor due to the non-uniform crack opening stress such as bending loads or other stress concentrations.

**Figure 1:**
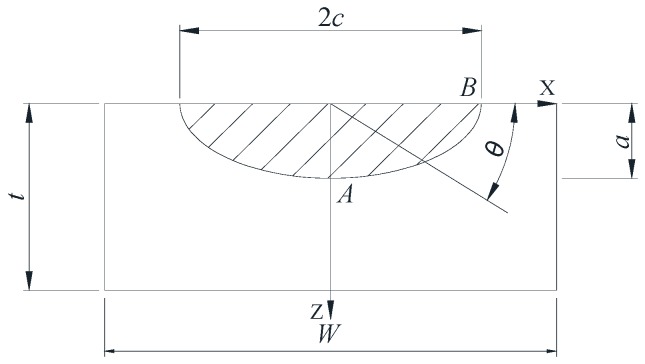
Semi-elliptical surface crack in a rectangular plate.

Based on the work of Albrecht and Yamada [34] and the hybrid method, Baik et al. [35] proposed a SIF equation for welded joints under bending stress. Equations (2) and (3), show the two equations used in the Baik-Yamada formula to calculate the SIF values at point *A* and point *B* (Figure 1), respectively [31].

(2) $$K_{A}=F_{GA}F_{sA}F_{T}F_{EA}F_{B}f\sigma_{b}\sqrt{\pi a}$$

(3) $$K_{B}=F_{GB}F_{sB}F_{W}F_{EB}F_{B}f\sigma_{b}\sqrt{\pi a}$$

where the physical meanings of *F_s_*, *F_W_*, *F_T_* and *F_E_* are the same as those in Equation (1); subscripts *A* and *B* represent point *A* and point *B*; *F_B_* and *f* are two new correction factors that account for the stress gradient due to the bending stress and the shift of the neutral axis as the crack propagates, respectively. The formula has the following two parts: One is the empirical equation for the SIF of a surface semi-elliptical crack on a rectangular plate and the other is the *F_G_* factor which reflects the non-uniform stress due to the local stress concentration of the weld. 

### 2.1. Comparison of the Baik-Yamada Formula with the Experimental Data and Benchmark Results

In the hybrid method, every correction factor in the Baik-Yamada formula, such as *F_s_*, *F_W_*, *F_T_*, and *F_E_*, represents a clear physical meaning. However, very few discussions have been carried out regarding whether these correction factors are appropriate. Xiao et al. [18] noted that, with the same parameters *C* and *m* in the Paris law and the same *F_G_* factor, the fatigue strength evaluation result obtained by the Baik-Yamada formula was worse than the result obtained by the Raju-Newman formula.

**Figure 2:**
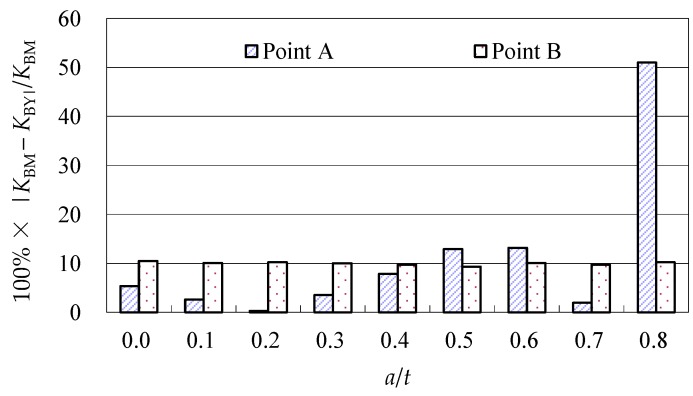
The Baik-Yamada formula vs. the benchmark results (*a*/*c* = 0.5).

The SIF values predicted by the Baik-Yamada formula were compared with the benchmark results [36] in Figure 2. As shown in Figure 2, although the differences at point *A* varied with different *a*/*t* ratios, the overall accuracy was acceptable for all the differences lower than 10% except when *a*/*t* = 0.8. When *a*/*t* = 0.8, the SIF at point *A* predicted by the Baik-Yamada formula equaled −0.039, whereas the benchmark result was 0.08. The differences at point *B* were relatively constant with different *a*/*t* ratios, and all the differences were around 10%. It should be noted that the differences at points *A* and *B* can be neglected when *a*/*t* is larger than 0.8. This is because, in the engineering application, the crack propagation rate will be too fast to be considered when *a*/*t* is beyond 0.8.

Table 1 shows the SIF values at point *A* from the experimental results [37] and the SIF values predicted by the Baik-Yamada formula. It is obvious that, with the increase of *a/t* and the decrease of *a/c*, the accuracy of the Baik-Yamada formula decreases. The difference is even larger than 50% when *a*/*t* is beyond 0.6.

**Figure 3:**
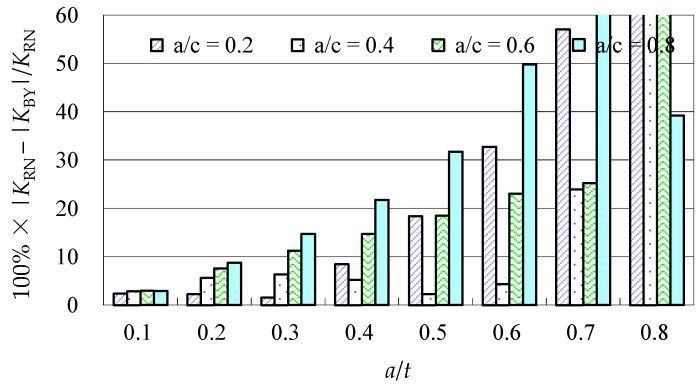
The Baik-Yamada formula vs. the Raju-Newman solution at point *A* (*c*/*W* = 0.1).

**Figure 4:**
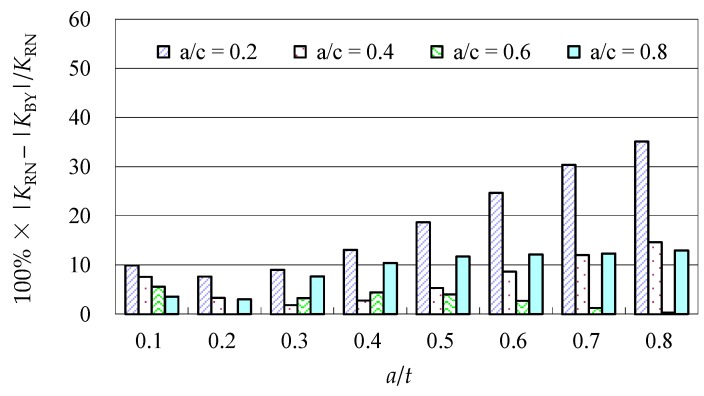
The Baik-Yamada formula vs. the Raju-Newman solution at point *B* (*c*/*W* = 0.1).

**Figure 5:**
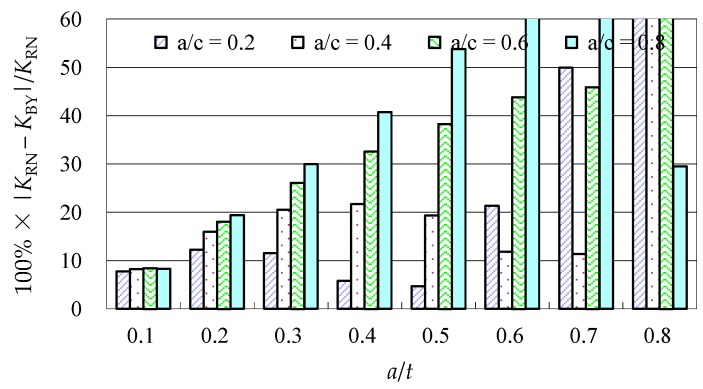
The Baik-Yamada formula vs. the Raju-Newman solution at point *A* (*c*/*W* = 0.2).

**Figure 6:**
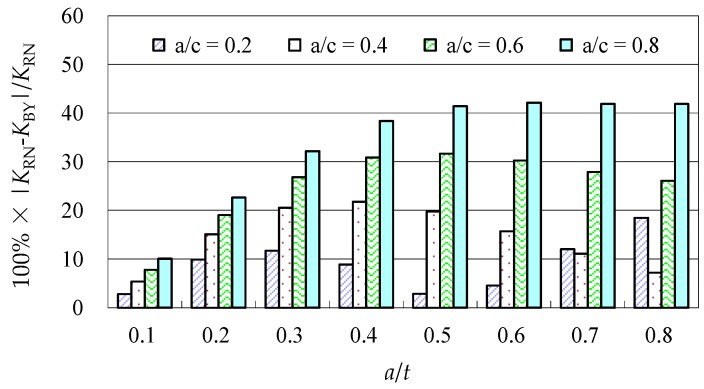
The Baik-Yamada formula vs. the Raju-Newman solution at point *B* (*c*/*W* = 0.2).

Due to the limited data from the benchmark results and experimental data, further evaluations of the accuracy of the Baik-Yamada formula need to be carried out. It is generally believed that the Raju-Newman formula provides accurate SIF results for surface cracks on a rectangular plate. Therefore, the accuracy of the Baik-Yamada formula is further verified using the predictions of the Raju-Newman formula against various *a*/*c* ratios, and these differences are shown in Figure 3, Figure 4, Figure 5 and Figure 6.

It was found in Figure 3 that, for different shape parameters, the differences at point *A* increased with the development of crack depth. When *a*/*t* is beyond 0.5, the differences are almost greater than 20% for various *a*/*c* values. In contrast, the differences at point *B* in Figure 4 decreased first before increasing with the *a*/*t* ratio. Except for *a*/*c* = 0.2, the difference for other *a*/*c* values is approximately 10%. In addition, when the *c/W* ratio increases from 0.1 to 0.2, the differences at point *A* and point *B* increase significantly. In Figure 5, the differences are generally larger than those in Figure 3 at the same *a*/*t* ratio. In Figure 6, the differences at point *B* are already approximately 30% for most *a*/*t* ratios. It is worth mentioning that, at point *A*, when *a*/*t* is close to 0.8, SIF tends to be zero or, even negative by the prediction using the Baik-Yamada formula. This is the reason why the difference becomes much larger when *a*/*t* is approximately 0.8, as shown in Figure 3 and Figure 5.

### 2.2. Reasons for the Low Accuracy of the Baik-Yamada Formula

#### 2.2.1. Lack of the Necessary Correction Factors for Points *A* and *B*

A comparison of Equations (1) and (2), found that the *F_W_* factor was absent from the equation for the SIF at point *A*. *F_W_* is the reflection of the finite width effect, and the absence of the *F_W_* factor may be one of the reasons for the low accuracy of the SIF prediction at point *A* as shown in Figure 3 and Figure 5.

A comparison of Equations (1) and (3), showed the absence of the *F_T_* factor from Equation (3) at point *B*. The *F_T_* factor is the reflection of the finite thickness effect. If the *f* factor were excluded from Equation (3) (further discussed in detail in Section 2.2.3), there would be no other factors in Equation (3) to reflect the finite thickness effect. For the bending stress, even at point *B*, the increase of *a*/*t* certainly causes the unstable calculation results, if no correction factor reflects the finite thickness effect on the SIF calculation. 

#### 2.2.2. Incomplete Equation of the *F_T_* Factor

Equation (4) shows the calculation of the *F_T_* factor at point *A*. It is a simple equation of *a*/*t*, and the effect of *a*/*c* is not considered. The *F_T_* factor represents the back surface correction [34], and it always dependent on the crack geometry, such as *a*/*c*, *a*/*t* and the bending stress [19]. Therefore, the current incomplete equation of the *F_T_* factor in Equation (2) causes reduced accuracy at point *A*, which tends to increase with the development of the crack depth *a*, as shown in Figure 3 and Figure 5. Therefore, a new regression equation of the *F_T_* factor needs to be developed by considering *a*/*c*, *a*/*t* and the bending stress.

(4) $$F_{T}=\sqrt{\sec\left(\pi a/2t\right)}$$

#### 2.2.3. Problem of the *f* Factor

**Figure 7:**
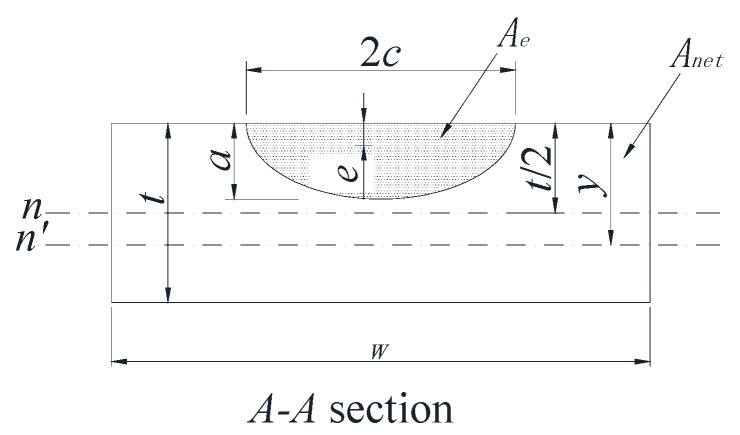
Neutral axis shift due to crack growth.

**Figure 8:**
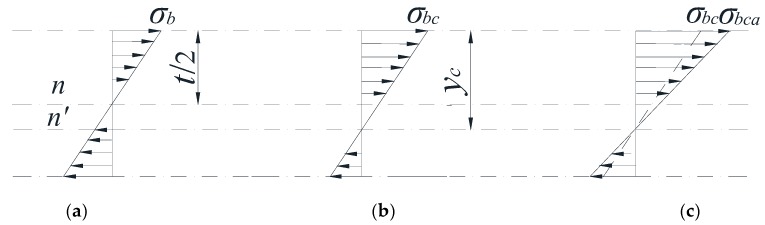
Stress diagrams for *A*-*A* section under bending stress (**a**) stress gradient without a neutral axis shift; (**b**) stress gradient with a neutral axis shift; (**c**) actual stress gradient with a neutral axis shift.

Baik et al. [35] proposed that crack growth gradually reduced the uncracked area in a rectangular plate. The reduction of the uncracked area causes the shift of the neutral axis of the section (which is the *A-A* section in Figure 1), as shown in Figure 7. In Figure 8, the initial bending stress σ*_b_* increases to σ*_bc_* due to the downward shift of the neutral axis. To correct the increment of the bending stress due to the downward shift of the neutral axis, σ*_bc_* is calculated and normalized against σ*_b_*. The *f* factor is defined in Equation (5), which reflects the growth range from σ*_b_* to σ*_bc_* [35].

(5) $$f=\frac{\sigma_{bc}}{\sigma_{b}}=\frac{Wt^{2}}{6I_{net}}y_{c}$$

where *y_c_* is the distance to the neutral axis *n*′; and *I_net_* is a moment of inertia of the uncracked area. Figure 8 shows three different stress gradients for the *A-A* section under bending stress. Figure 8a shows the normal stress gradient without a neutral axis shift. Figure 8b expresses the stress gradient with the neutral axis shift in the Baik et al. [35] paper. Figure 8c shows the actual stress gradient by the Baik-Yamada formula with the neutral axis shift and is calculated from Equation (5). 

However, for the *f* factor, both the physical concept and the calculation results have inevitable problems. It is widely accepted that in linear elastic fracture mechanics, there always exists a stress singularity at the crack tip, which means the neutral axis cannot be a straight line. Therefore, the assumption that the neutral axis is always a straight line is questionable.

Moreover, the calculation results of the *f* factor also have a certain limitation where the *c*/*W* cannot be large when the Baik-Yamada formula is used to calculate the SIF. As shown in Figure 8, the growth rate of σ*_bc_* from σ*_b_* always seems to be the same as the growth rate of the neutral axis from *t/*2 to *y_c_* [35]. When the *c*/*W* ratio is small, the increase rate of the bending stress mentioned above is valid because the change of *I_net_* is so small that it can be neglected. However, when *c*/*W* becomes larger, such as 0.15 or 0.2, from the Baik-Yamada formula results, the increase rate of σ*_bc_* from σ*_b_* depends not only on the variation range from *t/*2 to *y_c_* but also on the decreasing range of *I_net_*, for the reason that the change of *I_net_* cannot be ignored. 

As shown in Figure 8a,c, the actual change range from σ*_b_* to σ*_bca_* by the Baik-Yamada formula calculation is not proportional to the axis shift from *t/*2 to *y_c_*. The stress variation procedure is certainly not constant, which means that Figure 8b in the Baik and Yamada paper [35] is not correct. This is the main reason why the difference becomes much larger when *c/W* changes from 0.1 to 0.2, as shown in Figure 5 and Figure 6. Therefore, the Baik-Yamada formula will yield increased inaccuracy with the increase of *c/W*. In other words, the accuracy of the Baik-Yamada formula will decrease with the increase of *a*/*t* and the decrease of *a*/*c* when the thickness and width of the rectangular plate remain constant. This is also the key reason as to why the difference between the Baik-Yamada formula results and the photo-elastic experimental data becomes larger with larger cracks in Table 1.

In conclusion, due to several improper correction factors in the Baik-Yamada formula, the accuracy of the SIF calculation is not high enough. The Baik-Yamada formula needs to be modified with proper correction factors whose physical meanings are clearer, so that the application range can be extended.

## 3. Improved Stress Intensity Factor Formula Based on Theoretical Analysis and Numerical Regression

### 3.1. Deducing the F_T_ Factor Based on the Analytical Solution

Based on the reasons underlying the unsatisfactory accuracy of the SIF calculation mentioned in Section 3.2, there are three corresponding steps in which to modify the Baik-Yamada formula. The first step is to exclude the *f* factor, due to its unsuitable physical meaning and limitation in considering the *c*/*W* values. The second step is to add the *F_W_* factor and the *F_T_* factor shown in Equations (2) and (3), respectively, so that the physical meaning of each equation becomes complete. The same equation for the *F_W_* factor in Equation (3) can be used in Equation (2); however, the *F_T_* factor depends on the crack geometry and the bending stress. Therefore, the third step is to determine the *F_T_* factor and ensure that no other physical factors will affect the SIF value under bending stress.

As mentioned in Section 2.1, the Baik-Yamada formula is based on the hybrid method in which correction factors reflect particular physical meanings of the different effects. The alternating approach provides analytical SIF solutions and referenced equations of the correction factors. Therefore, the determination of the *F_T_* factor and the identification of any other new correction factors should also be based on the analytical SIF solutions from the alternating approach.

By using the alternating method, the analytical SIF solution for elliptical cracks embedded in the approximate infinite solid under bending stress can be obtained [38]. Detailed SIF formula is given in Appendix A. When compared with the SIF due to remote tension stress, it is found that the analytical SIF solution due to the linearly varying stress contains a special part as shown in Equation (A3) (see Appendix A). Equation (A3) can be treated as a function of two parameters, θ and *a*/*c*.

Similarly, it is easy to establish the corresponding parameters in the Baik-Yamada formula. At point *A*, θ equals 90 degrees in Equation (A3). In this case, *F*(θ,*a/c*) becomes a function of *a/c* because both *K*(*k*) and *E*(*k*) are functions of *a*/*c*. This indicates that for bending stress, the parameter *a*/*c* has an effect on the SIF at point *A*. It is interesting to note that the *F_B_* factor in the Baik-Yamada formula illustrates the effect of *a*/*t* (in the depth direction) on the SIF under bending stress [35]. However, when the *f* factor is excluded, no factor in the Baik-Yamada formula reflects the effect of *a/c* (in the surface direction) on the SIF. Therefore, a factor is necessary for the Baik-Yamada formula that can reflect the effect of *a/c* on the SIF under bending stress. In addition, the *F_T_* factor in Equation (3) also needs to be modified so that it can reflect the effect of *a*/*c* on the SIF. Therefore, for the purpose of simplification, the new correction factor under bending stress can be combined with the modified *F_T_* factor at point *A*, in the form of *F_TA_* = *F*(*a/t*, *a/c*). The modified Baik-Yamada formula at point *A* can be expressed as

(6) $$K_{A}=F_{GA}F_{sA}F_{TA}F_{W}F_{EA}F_{B}\sigma_{b}\sqrt{\pi a}$$

where the expressions of *F_s_*, *F_E_*, *F_B_*, *F_W_* and *F_GA_* are the same as those in the Baik-Yamada formula in Equation (2).

At point *B*, θ equals zero degrees in Equation (A3). In this case, *F*(θ,*a/c*) becomes zero which means that *a/c* actually has no effect on the SIF at point *B* under bending stress. A similar conclusion was made by Shah and Kobayashi [38] that indicated that the bending stress had little impact on the SIF at point *B*. In addition, the *F_B_* factor in Equation (6), which presents the effect of the stress gradient under bending stress on the SIF, equals one, which further proves that *a/c* has no effect on the SIF at point *B*. Therefore, the modification of the Baik-Yamada formula at point *B* only needs to add the *F_TB_* factor in Equation (3) and exclude the *f* factor, as shown in Equation (7):

(7) $$K_{B}=F_{GB}F_{sB}F_{TB}F_{W}F_{EB}F_{B}\sigma_{b}\sqrt{\pi a}$$

In addition, the physical meaning of the correction factors in Equations (6) and (7) can be verified by the alternating approach. The SIF of a semi-elliptical surface crack in a plate subjected to pure bending can also be estimated by the alternating approach [38]. The formula can be divided into the following two parts: the first part is the SIF of a semi-elliptical surface crack in a rectangular plate subjected to uniform tensile stress, and the second part is the SIF of an elliptical crack in a semi-infinite solid subjected to linearly varying stress.

The SIF equation at point *A* (Equation (A1)) adds one more term $F_{LA}\frac{Ma}{I}(1+R)$, compared to the SIF at point *B* (Equation (A2)). Obviously, this term is a function of *a/t* and *a/c*. *F_B_* reflects the effect of *a/t* and *F_TB_* the effect of *a/c* on the SIF.

By comparing Equation (A2) with Equation (7), it is easy to find that part $\frac{\sqrt{a/c}}{E(k)}$ corresponds to the *F_EB_* factor. *R* is not included in order to exclude the effect of the bending stress on the SIF for point *B*. $F_{KB}\left(\frac{Mt}{2I}+\frac{Ma}{I}\right)-F_{LB}\frac{Ma}{I}$ corresponds to bending stress and related correction factors, such as *F_sB_*, *F_W_*, and *F_TB_*. It is noticed that the value of $F_{KB}\left(\frac{Mt}{2I}+\frac{Ma}{I}\right)-F_{LB}\frac{Ma}{I}$ changes with *a*/*c*, meaning that *a*/*c* has an effect on the SIF at point *B*. Meanwhile, *F_sB_* and *F_W_* do not contain the parameter *a/t*, indicating that even at point *B*, the *F_TB_* factor is necessary for the SIF equation.

### 3.2. Development of the F_T_ Factor using Finite Element Analysis 

The modified Baik-Yamada formula was clarified and every correction factor was determined with the exception of the new *F_T_* factor. In this section, the relevant SIF values are achieved using the finite element (FE) method so that the new *F_T_* factors at points *A* and *B* can be derived using the FE results.

**Figure 9:**
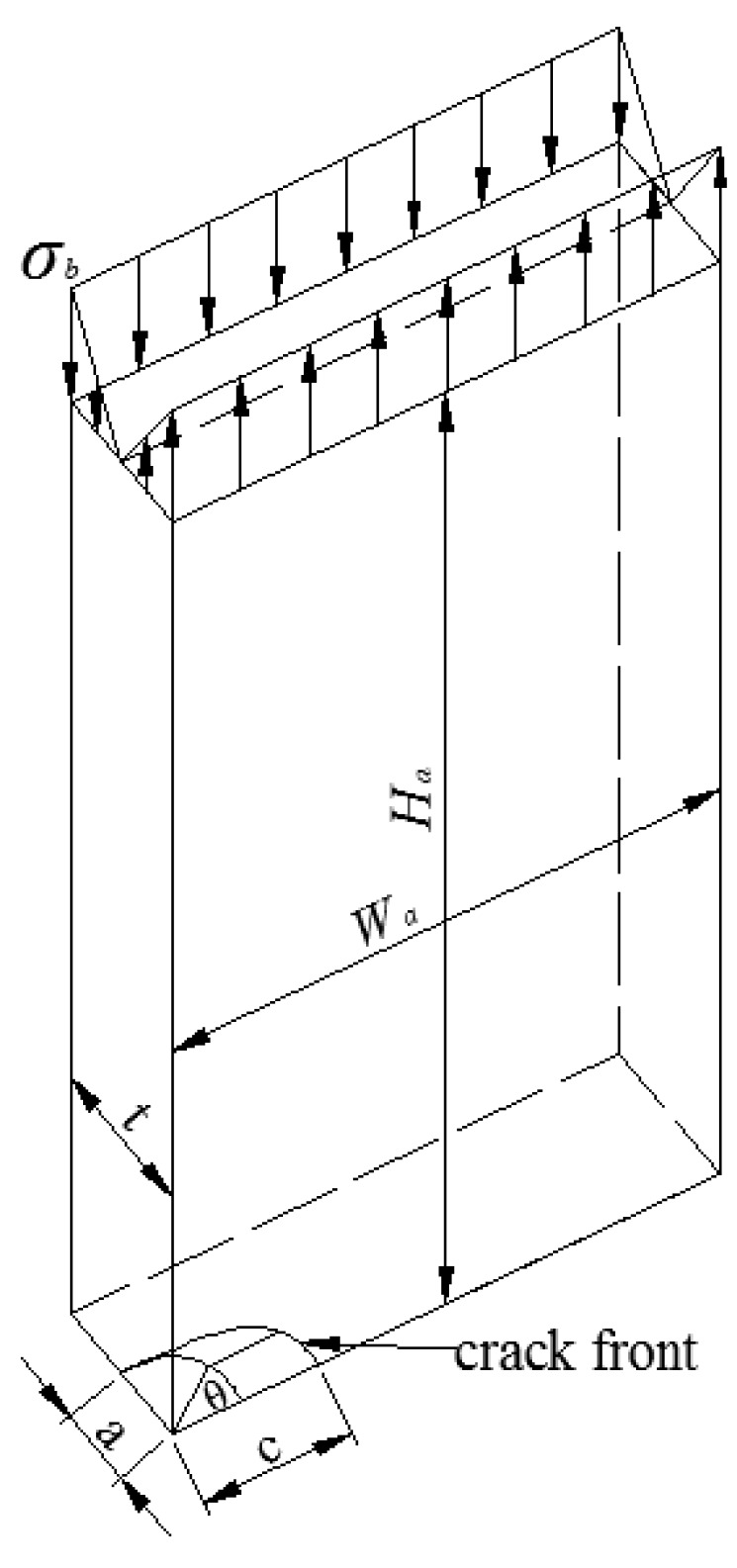
One-quarter model for the semi-elliptical crack in a rectangular plate.

Finite element software ABAQUS was adopted for the FE simulation. The geometry of the semi-elliptical crack in a rectangular plate is shown in Figure 9. Considering the symmetry of the boundary condition and the geometry of the body, only a quarter of the rectangular plate was considered in the FE simulation with *W* = 10 mm and *H* = 10 mm. The plate was subjected to bending stress, and the material used was structural steel with a Young’s modulus of 210 GPa and a Poisson’s ratio of 0.3. The element of the plate was C3D8R.

To reveal the geometric characteristics of the crack in the rectangular plate, two dimensionless parameters (*a*/*t* and *a*/*c*) were selected. Eleven values of *a*/*t* were considered in this FE study as follows: 0.1, 0.2, 0.3, 0.4, 0.5, 0.6, 0.7, 0.72, 0.75, 0.78 and 0.8. The cases with *a*/*t* higher than 0.8 were not considered in the current study as the cracks would have grown too fast in engineering practice. Meanwhile, in the Baik-Yamada formula, the *F_B_* decreased at point *A* with the increase of *a*/*t* and became zero at approximately *a*/*t* = 0.78 [35]. In order to ensure the stability and continuity of the data at point *A*, three values (0.72, 0.75, 0.78) of *a*/*t* were chosen between 0.7 and 0.8. The SIF was not very sensitive to *a*/*c* [39]. Therefore, the following five values of *a*/*c* were selected: 0.2, 0.4, 0.6, 0.8 and 1.0. 

**Figure 10:**
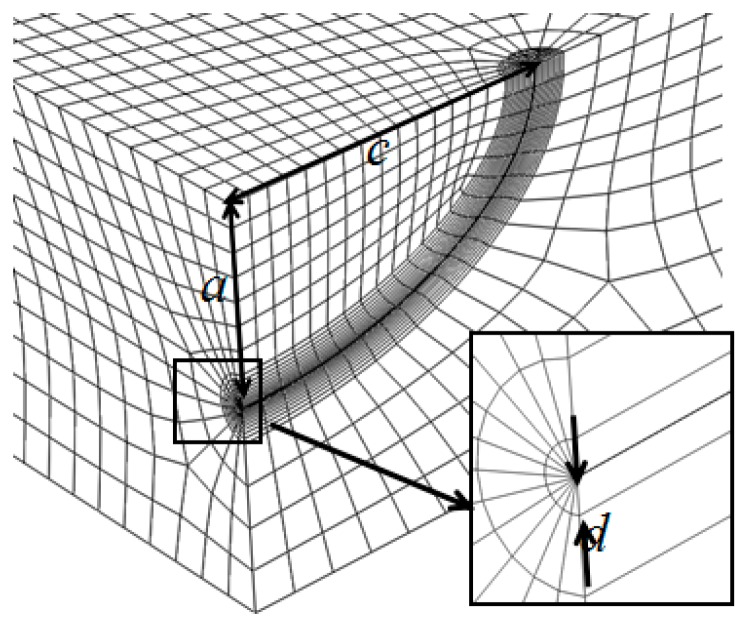
The singular element mesh with connected conventional elements around the crack tip.

To build a suitable mesh for the FE model, two characteristics of the FE model need to be considered. The first is the singularity at the region around the crack tip. The second involves the boundary effects caused by the crack. To address the first issue, three-dimensional wedge singular elements were attached to the crack tip. Figure 10 shows the details of the mesh in the region close to the three-dimensional semi-elliptical crack with the element size of *d* = 0.005 mm. As shown in Figure 10, a fine mesh was used for the region within 0.02 mm from the crack tip. A coarse mesh was adopted for the regions that were further away from the crack. In Figure 10, all elements along the crack tip were singular elements. To address the second issue of the FE mesh, Saint-Venant’s principle was used where the stress distribution can be deemed as not being affected by the crack, if the point of interest is sufficiently far from the crack. The crack width ratio *c*/*W* and the crack height ratio *c*/*H* were kept constant and were equal to 0.1. 

The SIF results by the FE simulation were first validated using the Raju-Newman formula because of its widely accepted accuracy. Next, the new *F_T_* factor at both points *A* and *B* were obtained using the validated FE model. The expression of the new *F_T_* factors derived by the data regression method are demonstrated in the next section, along with the complete modified Bail-Yamada formula.

**Figure 11:**
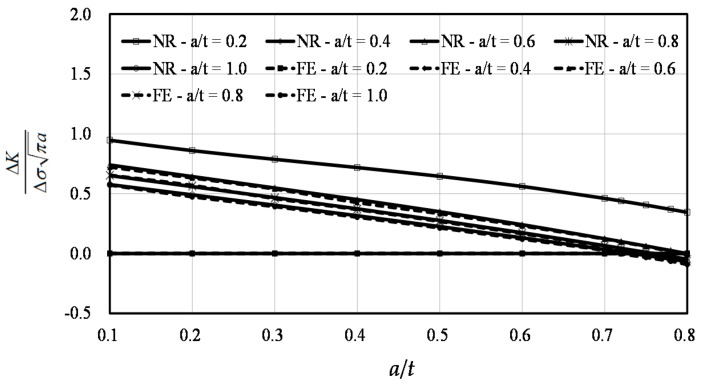
The finite element results vs. the Raju-Newman solution at point *A*.

**Figure 12:**
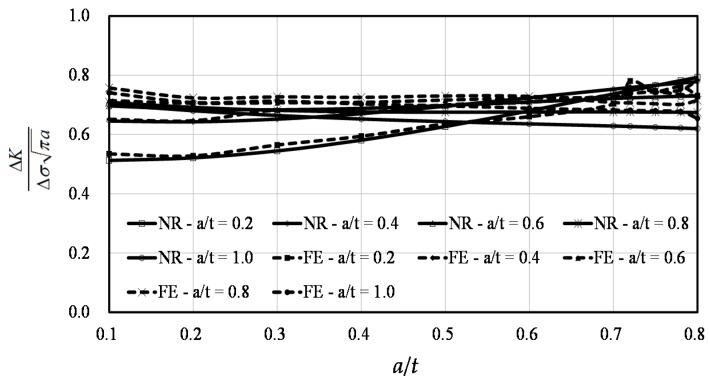
The finite element results vs. the Raju-Newman solution at point *B*.

The comparisons between the predictions by the Raju-Newman formula and the FE results at points *A* and *B* are shown in Figure 11 and Figure 12, respectively. In these two figures, FE indicates the results of the finite element simulation and RN represents the results of the Raju-Newman solution.

A total of 55 calculations were performed at point *A,* including three values of *a*/*t* between 0.7 and 0.8. The FE results agreed well with the predictions made by the Raju-Newman formula, as shown in Figure 11. When the dimensionless SIF values were beyond 0.1, the average difference at point *A* was 4.06% and the maximum difference was under 10%. The reason as to why the difference was not included when the SIF was under 0.1, was that several pieces of data were too small that the proportional difference was meaningless.

40 calculations were conducted at point *B*, without considering the three values of *a*/*t* at 0.72, 0.75, and 0.78 for simplification purposes. As shown in Figure 12, the FE results agreed well with the results from the Raju-Newman formula. The average difference was only 4.56% and the maximum difference was under 9%, indicating the reliability of the FE results. 

### 3.3. Improved Stress Intensity Factor Formula under Bending Stress

Except for the new *F_T_* factor, other factors are known in the Baik-Yamada formula. The SIF values have been obtained from FE simulations. Therefore, the new *F_T_* factor values for point *A* and point *B* can be achieved by dividing the SIF by the other known correction factors in Equations (6) and (7).

At point *A*, 43 values of the new *F_T_* factor were calculated for regression purposes. These values corresponded to *a*/*t* at 0.1, 0.2, 0.3, 0.4, 0.5, 0.6, 0.7, 0.72, and 0.75 when the *a*/*c* ranged from 0.2 to 0.8; and *a*/*t* at 0.1, 0.2, 0.3, 0.4, 0.5, 0.6, and 0.7 when *a*/*c* equaled one. It should be noted that *a*/*t* at 0.78 and 0.80 were not considered when *a*/*c* ranged from 0.2 to 0.8 because the *F_B_* factor became zero when *a*/*t* = 0.78, which made the new *F_T_* factor meaningless. In addition, *a*/*t* larger than 0.7 were not considered when the *a*/*c* was equal to one because the SIF became negative when *a*/*t* exceeded 0.7. Finally, the new *F_TA_* factor for point *A* was obtained through data regression as shown in Equation (8):

(8) $$\begin{array}{l} F_{TA}=(1.46\frac{a}{t}-1.20\frac{a}{c}+1.40){\left[2.22{\left(\frac{a}{c}\right)}^{2}-4.59\frac{a}{c}\frac{a}{t}+1.56{\left(\frac{a}{t}\right)}^{2}+0.69\right]}^{1.05}\\ \times {\left[\frac{a}{c}-0.53{\left(\frac{a}{t}\right)}^{2}+0.90\frac{a}{c}{\left(\frac{a}{t}\right)}^{2}\right]}^{-9.96{\left(\frac{a}{c}\right)}^{2}{\left(\frac{a}{t}\right)}^{2}} \end{array}$$

In the surface direction, 40 values of the new *F_T_* factor were obtained for regression purposes. These values corresponded to *a*/*t* at 0.1, 0.2, 0.3, 0.4, 0.5, 0.6, 0.7, and 0.8 when *a*/*c* ranged from 0.2 to 1.0. Three values of *a*/*t* between 0.7 and 0.8 were excluded because the SIF for point *B* became less sensitive. The new *F_TB_* factor for point *B* was also obtained through the data regression method, as shown in Equation (9):

(9) $$\begin{array}{l} F_{TB}=(0.14\frac{a}{t}-0.88\frac{a}{c}+1.22){\left[0.28{\left(\frac{a}{c}\right)}^{2}-0.30\frac{a}{c}\frac{a}{t}-0.06{\left(\frac{a}{t}\right)}^{2}+1.02\right]}^{4.65}\\ \times {\left[\frac{a}{c}+7.03{\left(\frac{a}{t}\right)}^{2}+\frac{a}{c}\frac{a}{t}\right]}^{0.44\frac{a}{t}} \end{array}$$

Finally, the modified Baik-Yamada formula for the SIF at point *A* and point *B* was expressed as Equations (6) and (7), respectively. The complete equations are as follows:

(10) $$K_{A}=F_{GA}\left[1.12-0.12(\frac{a}{c})\right]F_{TA}\sqrt{\sec(\frac{\pi c}{W})}\frac{1}{\sqrt{1+1.464{\left(a/c\right)}^{1.65}}}(1-\frac{4a}{\pi t})\sigma_{b}\sqrt{\pi a}$$

(11) $$K_{B}=F_{GB}\times 1\times F_{TB}\sqrt{\sec(\frac{\pi c}{W})}\sqrt{\frac{a/c}{1+1.464{\left(a/c\right)}^{1.65}}}(1-\frac{4a}{\pi t})\sigma_{b}\sqrt{\pi a}$$

## 4. Validation

### 4.1. The Stress Intensity Factor of a Semi-Elliptical Crack in the Rectangular Plate

For validation purposes, the SIF results predicted by the modified formula in Equation (10) and Equation (11) were compared with SIF data from the literature, including benchmark results [36], photo-elastic experimental data [37], Raju-Newman formula [28] and three-dimensional FE results for welded joints [17]. It was found that the accuracy of the SIF from the modified formula was much better than that calculated by the Baik-Yamada formula.

**Figure 13:**
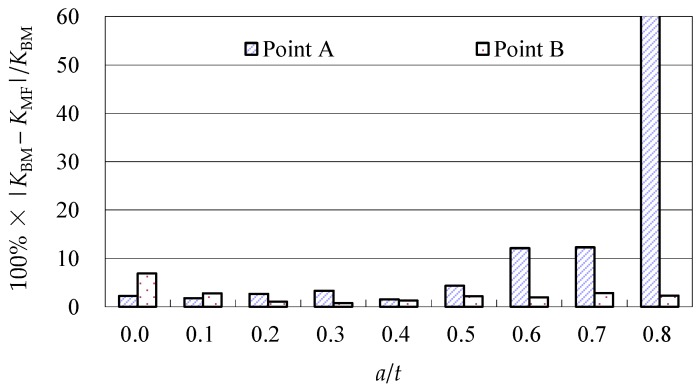
Modified formulae vs. the benchmark results (*a*/*c* = 0.5).

The comparison of the SIF from the modified formula with the benchmark results is shown in Figure 13. Compared with Figure 2, the accuracy of the modified formulae was increased, especially at point *B*. The difference was lower than seven percent and the average difference was only 3.4%. At point A, most differences were under five percent except for *a/t* at 0.6, 0.7 and 0.8. The difference was slightly higher than 10% at an *a/t* of 0.6 and 0.7 due to the small SIF values. When *a/t* = 0.8, the SIF was negative using the modified formulae (−0.05), and should not be accounted for in the accuracy evaluation.

Table 2 shows the SIF values at point *A* from the modified formula compared with the photo-elastic experimental data. Compared to Table 1, it is obvious that the modified formula has improved accuracy. The difference of the results from the modified formula is approximately 25%. Mahmoud and Hosseini [40] found that the maximum difference between the photo-elastic experimental results and the Raju-Newman formula was 56% and was much larger than the difference shown in Table 2.

**Figure 14:**
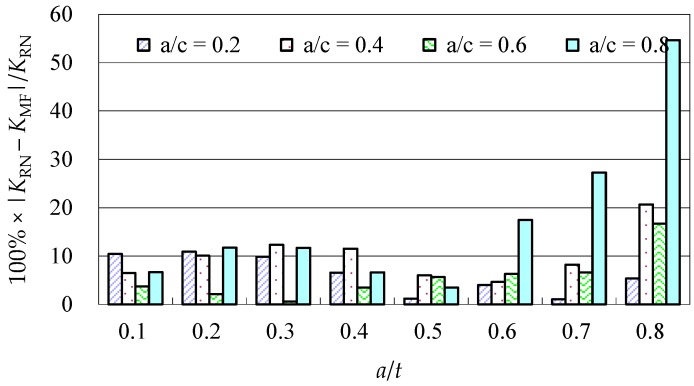
Modified formula vs. the Raju-Newman solution at point *A* (*c*/*W* = 0.1).

**Figure 15:**
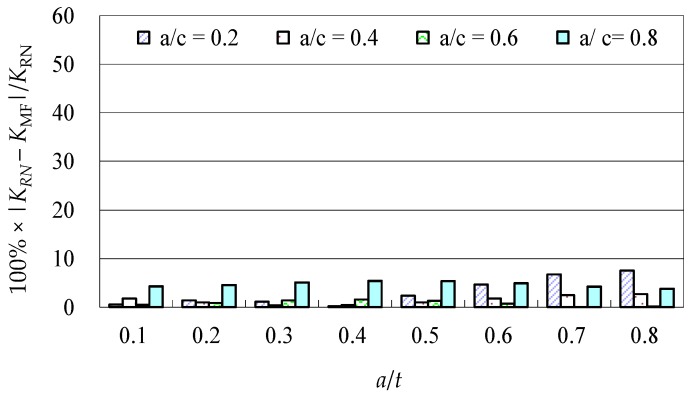
Modified formula vs. the Raju-Newman solution at point *B* (*c*/*W* = 0.1).

**Figure 16:**
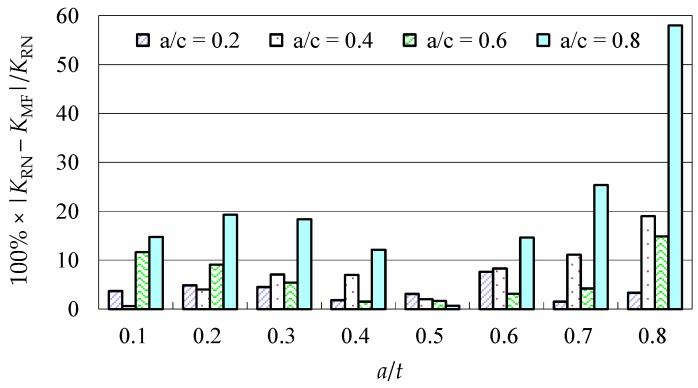
Modified formula vs. the Raju-Newman solution at point *A* (*c*/*W* = 0.2).

**Figure 17:**
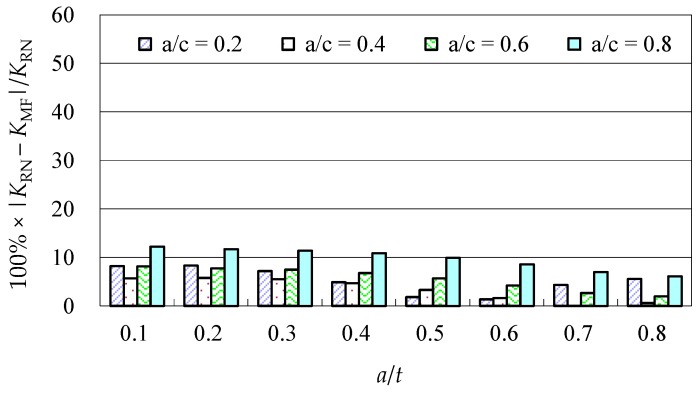
Modified formula vs. the Raju-Newman solution at point *B* (*c*/*W* = 0.2).

A comparison of the SIF from the modified formula and the Raju-Newman formula is shown in Figure 14, Figure 15, Figure 16 and Figure 17. It should be noted that the maximum value of *a*/*t* was chosen to be 0.75 based on the data regression process in Section 3.3, where the terminal point for *a*/*t* at point *A* was chosen to be 0.75 to avoid the singularity problem. Compared to Figure 3, Figure 4, Figure 5 and Figure 6, the modified formula is more accurate than the Baik-Yamada formula. As shown in Figure 14 and Figure 16, most of the differences of the modified formula for the depth point are approximately 10% for *c*/*W* at 0.1 and 0.2. When *a*/*t* is beyond 0.5, the difference becomes larger, especially for *a*/*c* at 0.6 and 0.8. This is because the actual SIF values under certain conditions are small (generally under 0.25), which causes the relatively large difference. At point *B*, all the differences in Figure 15 are lower than 10%. When *c*/*W* equals 0.2 as shown in Figure 17, the difference increases slightly but is still approximately 10%, which is much better than the case in Figure 6. 

By comparing the modified formulae and the existing research results, the accuracy of the modified formula for the rectangular plate is verified, which means that the accuracy for the welded joints can be ensured theoretically as long as the *F_G_* factor is appropriate.

### 4.2. The Stress Intensity Factor of a Semi-Elliptical Crack in a T-Shaped Welded Joint

In addition, a recent three-dimensional FE study on the SIF of a T-shaped welded joint was reported in Reference [17]. The SIF results in Reference [17] were used to further validate the accuracy of the modified formula. Three-dimensional finite element method (3D FEM), wavelet and extended finite element method (WX-FEM) and the empirical formulae from BSI fatigue document (BS 7910) [25] were employed in Reference [17] to calculate the SIF values. The maximum bending stress applied was 200 MPa. The plate width was 100 mm. The footprint width of the rib was 27.5 mm and the main plate thickness was 22 mm. The relative SIF data was not explicitly shown in Reference [17]. The SIF data of interest could only be obtained by reading the crack propagation figures, and the SIF data are presented in Table 3, Table 4, Table 5, Table 6, Table 7 and Table 8. It should be noted that the range of *a*/*t* was not as large as the ranges of *a*/*c* and *c/W, which* are larger. Therefore, the accuracy of the modified formula can be further validated using the results in Reference [17]. In these tables, the dimensionless SIF values of *F_A_* and *F_B_* are calculated by the following equations:

(12) $$F_{A}=\frac{K_{A}}{\sigma_{b}\sqrt{\pi a}}$$

(13) $$F_{B}=\frac{K_{B}}{\sigma_{b}\sqrt{\pi a}}$$

When using the modified formula to calculate the SIF of the welded joints, the *F_G_* factor is necessary. The acquisition process of the *F_G_* factor is briefly described as follows. The stress distribution at the welded toe without the crack can be obtained by FE simulation. Then, the *F_GA_* factor values at each point can be obtained by following the method proposed by Albrecht and Yamada [34]. *F_GB_* takes a constant value at *a* = 0.1 mm [18].

The comparisons of the dimensionless SIF values from the Baik-Yamada formula, the modified Baik-Yamada formula and the results in Reference [17] are shown in Table 3, Table 4, Table 5, Table 6, Table 7 and Table 8. With the same *F_G_* value, the accuracy of the modified formula was much better than that of the Baik-Yamada formula. At point *A*, the difference of the Baik-Yamada formula was unstable, and the maximum value of the average difference was higher than 50%. However, the maximum difference for the modified formula at point *A* was under 40%. At point *B*, the maximum value of the average difference from the Baik-Yamada formula was approximately 74% while the maximum value of the average difference from the modified formula was under 39%. This comparison shows that the modified formula can also be used for calculation of the SIF of the welded joints in engineering applications.

## 5. Conclusions

In this paper, the major causes of a reduced accuracy of the stress intensity factor from an empirical formula were investigated. Subsequently, corresponding correction factors in this formula were modified according to the analytical SIF formula. The finite element simulation was adopted for the regression of one important correction factor in the modified formula. Finally, the modified formula was validated by the experimental and numerical data in the literature. It was concluded that the proposed modified formula is reliable and accurate for the calculation of the SIF of a semi-elliptical surface crack in welded joints under bending stress.

## Figures and Tables

**Table 1:** The Baik-Yamada formula vs. the photo-elastic experimental data.

*a* (mm)	*c* (mm)	*a/c*	*a/t*	*c/W*	*K* from [37] (N/m^3/2^)	*K* from the BY Formula (N/m^3/2^)	Difference (%)
7.36	14.50	0.51	0.37	0.03	0.44	0.51	18.04
					0.43	0.51	15.75
13.10	41.65	0.31	0.62	0.09	0.48	0.21	61.28
					0.42	0.21	55.53

**Table 2:** Modified formulae vs. photo-elastic experiment data.

*a* (mm)	*c* (mm)	*a/c*	*a/t*	*c/W*	*K* from [37] (N/m^3/2^)	*K* from Mod. Formula (N/m^3/2^)	Difference (%)
7.36	14.50	0.51	0.37	0.03	0.44	0.48	19.96
					0.43	0.48	17.72
13.10	41.65	0.31	0.62	0.09	0.48	0.42	30.15
					0.42	0.42	19.79

**Table 3:** Comparison of the dimensionless stress intensity factor values from the Baik-Yamada formula, the modified formula and the literature results by the wavelet and extended finite elememt method at point *A*.

*a* (mm)	*c* (mm)	*a*/*t*	*a/c*	*c/W*	*F_A_* [17]	*F_A,BY_*	Difference (%)	*F_A,Mod._*	Difference (%)
0.10	0.10	0.005	1.00	0.001	1.83	1.55	15.26	0.98	46.65
1.10	1.17	0.050	0.93	0.012	0.87	0.81	6.43	0.72	16.99
1.97	2.55	0.090	0.77	0.026	0.77	0.75	2.59	0.80	2.91
3.07	3.93	0.139	0.78	0.039	0.69	0.66	3.68	0.71	2.58
4.10	5.40	0.186	0.76	0.054	0.63	0.62	2.24	0.65	2.89
5.08	7.20	0.231	0.71	0.072	0.59	0.61	2.30	0.61	2.94
6.17	9.28	0.281	0.67	0.093	0.56	0.59	4.99	0.56	0.85
7.10	11.53	0.323	0.62	0.115	0.53	0.59	9.62	0.51	3.89
8.04	14.38	0.366	0.56	0.144	0.51	0.59	14.95	0.48	6.87
9.18	18.11	0.417	0.51	0.181	0.49	0.60	21.50	0.45	7.69
10.12	22.04	0.460	0.46	0.220	0.48	0.61	26.87	0.46	5.61
10.95	25.77	0.498	0.43	0.258	0.47	0.63	33.75	0.48	0.78
						Mean	11.72	Mean	4.91

**Table 4:** Comparison of the dimensionless stress intensity factor values from the Baik-Yamada formula, the modified formula and the literature results by the wavelet and extended finite elememt method at point *B*.

*a* (mm)	*c* (mm)	*a*/*t*	*a/c*	*c/W*	*F_B_* [17]	*F_B,BY_*	Difference (%)	*F_B,Mod._*	Difference (%)
0.10	0.10	0.005	1.00	0.001	0.44	1.56	253.56	1.80	306.77
1.10	1.17	0.050	0.93	0.012	0.70	1.56	123.21	1.78	154.22
1.97	2.55	0.090	0.77	0.026	0.91	1.56	71.42	1.68	84.12
3.07	3.93	0.139	0.78	0.039	1.06	1.59	49.42	1.70	59.43
4.10	5.40	0.186	0.76	0.054	1.19	1.63	36.86	1.70	43.11
5.08	7.20	0.231	0.71	0.072	1.29	1.67	29.37	1.68	30.14
6.17	9.28	0.281	0.67	0.093	1.39	1.73	24.06	1.67	19.74
7.10	11.53	0.323	0.62	0.115	1.49	1.80	20.84	1.65	10.81
8.04	14.38	0.366	0.56	0.144	1.60	1.90	19.19	1.63	2.31
9.18	18.11	0.417	0.51	0.181	1.73	2.09	20.35	1.64	5.45
10.12	22.04	0.460	0.46	0.220	1.86	2.34	26.03	1.66	10.39
10.95	25.77	0.498	0.43	0.258	1.97	2.68	35.99	1.71	13.01
						Mean	41.52	Mean	39.34

**Table 5:** Comparison of the dimensionless stress intensity factor values from the Baik-Yamada formula, the modified formula and the literature results by the three-dimensional finite element method at point *A*.

*a* (mm)	*c* (mm)	*a*/*t*	*a/c*	*c/W*	*F_A_* [17]	*F_A,BY_*	Difference (%)	*F_A,Mod._*	Difference (%)
0.10	0.10	0.005	1.00	0.001	1.70	1.55	8.42	0.98	42.35
0.98	1.29	0.045	0.76	0.013	0.85	0.94	10.29	0.97	14.34
2.01	3.07	0.091	0.65	0.031	0.73	0.81	10.61	0.85	15.56
2.89	5.05	0.131	0.57	0.051	0.68	0.78	14.86	0.77	13.87
3.97	7.31	0.180	0.54	0.073	0.62	0.73	18.70	0.69	11.72
5.11	9.90	0.232	0.52	0.099	0.57	0.70	23.89	0.62	9.70
5.88	12.09	0.267	0.49	0.121	0.54	0.70	0.01	0.59	9.26
6.95	15.37	0.316	0.45	0.154	0.51	0.70	36.10	0.55	8.05
8.05	19.08	0.366	0.42	0.191	0.49	0.70	42.37	0.53	8.57
9.02	22.74	0.410	0.40	0.227	0.48	0.71	48.12	0.53	11.33
9.89	26.67	0.450	0.37	0.267	0.49	0.74	51.10	0.56	14.47
11.13	32.14	0.506	0.35	0.321	0.50	0.78	56.24	0.62	23.07
						Mean	28.39	Mean	12.72

**Table 6:** Comparison of the dimensionless stress intensity factor values from the Baik-Yamada formula, the modified formula and the literature results by the three-dimensional finite element method at point *B*.

*a* (mm)	*c* (mm)	*a*/*t*	*a/c*	*c/W*	*F_B_* [17]	*F_B,BY_*	Difference (%)	*F_B,Mod._*	Difference (%)
0.10	0.10	0.005	1.00	0.001	0.40	1.56	291.93	1.80	350.92
0.98	1.29	0.045	0.76	0.013	0.78	1.54	96.53	1.66	112.02
2.01	3.07	0.091	0.65	0.031	1.05	1.53	45.89	1.57	49.24
2.89	5.05	0.131	0.57	0.051	1.19	1.53	28.52	1.51	26.41
3.97	7.31	0.180	0.54	0.073	1.31	1.58	19.95	1.51	14.88
5.11	9.90	0.232	0.52	0.099	1.42	1.64	15.58	1.52	6.94
5.88	12.09	0.267	0.49	0.121	1.50	1.70	13.93	1.52	1.84
6.95	15.37	0.316	0.45	0.154	1.59	1.83	14.72	1.54	3.23
8.05	19.08	0.366	0.42	0.191	1.74	2.01	15.60	1.57	9.62
9.02	22.74	0.410	0.40	0.227	1.86	2.26	21.21	1.62	13.15
9.89	26.67	0.450	0.37	0.267	2.06	2.62	27.01	1.69	18.18
11.13	32.14	0.506	0.35	0.321	2.32	3.46	49.10	1.85	20.26
						Mean	31.64	Mean	25.07

**Table 7:** Comparison of dimensionless stress intensity factor values from the Baik-Yamada formula, the modified formula and the literature results of the empirical formula at point *A*.

*a* (mm)	*c* (mm)	*a*/*t*	*a/c*	*c/W*	*F_A_* [17]	*F_A,BY_*	Difference (%)	*F_A,Mod._*	Difference (%)
0.10	0.10	0.005	1.00	0.001	1.55	1.83	15.26	0.98	46.65
0.96	1.60	0.044	0.60	0.016	1.04	0.98	7.05	1.08	10.89
1.98	3.62	0.090	0.55	0.036	0.87	0.82	6.27	0.87	6.30
3.04	6.28	0.138	0.48	0.063	0.82	0.74	11.02	0.76	3.86
4.05	9.36	0.184	0.43	0.094	0.80	0.67	18.50	0.70	3.52
4.97	12.29	0.226	0.40	0.123	0.78	0.63	23.88	0.65	2.98
5.93	16.06	0.270	0.37	0.161	0.79	0.60	32.51	0.63	5.31
7.10	21.01	0.323	0.34	0.210	0.81	0.56	43.71	0.63	10.77
8.11	26.23	0.369	0.31	0.262	0.86	0.55	56.00	0.66	19.84
9.07	31.50	0.412	0.29	0.315	0.93	0.55	70.15	0.73	32.89
10.19	39.26	0.463	0.26	0.393	1.15	0.58	97.64	0.95	64.08
11.35	48.47	0.516	0.23	0.485	1.88	0.66	183.83	2.52	280.44
						Mean	50.05	Mean	40.08

**Table 8:** Comparison of dimensionless stress intensity factor values from the Baik-Yamada formula, the modified formula and the literature results of the empirical formula at point *B*.

*a* (mm)	*c* (mm)	*a*/*t*	*a/c*	*c/W*	*F_B_* [17]	*F_B,BY_*	Difference (%)	*F_B,Mod._*	Difference (%)
0.10	0.10	0.005	1.00	0.001	0.64	1.56	145.17	1.80	182.07
0.96	1.60	0.044	0.60	0.016	0.94	1.49	59.32	1.52	61.91
1.98	3.62	0.090	0.55	0.036	1.18	1.49	26.17	1.47	24.69
3.04	6.28	0.138	0.48	0.063	1.36	1.50	10.42	1.44	5.88
4.05	9.36	0.184	0.43	0.094	1.51	1.53	1.89	1.43	5.35
4.97	12.29	0.226	0.40	0.123	1.59	1.60	0.65	1.44	9.70
5.93	16.06	0.270	0.37	0.161	1.72	1.71	0.16	1.46	15.07
7.10	21.01	0.323	0.34	0.210	1.88	1.95	3.36	1.51	19.62
8.11	26.23	0.369	0.31	0.262	2.05	2.31	12.71	1.60	22.01
9.07	31.50	0.412	0.29	0.315	2.23	2.94	31.51	1.75	21.76
10.19	39.26	0.463	0.26	0.393	2.61	4.94	88.82	2.18	16.53
11.35	48.47	0.516	0.23	0.485	3.37	22.92	579.88	5.48	62.70
						Mean	74.08	Mean	24.11

## Appendix A. Analytical Solution of SIF for Rectangular Plate under Bending Stress

Shah and Kobayashi deduced the SIF equations for point *A* and point *B* (Figure 1) of the semi-elliptical crack of the rectangular plate under bending stress [38]. The SIF equations are expressed in Equations (A.1) and (A.2), respectively.

(A1) $$K_{A}=\left[F_{KA}\left(\frac{Mt}{2I}+\frac{Ma}{I}\right)-F_{LA}\frac{Ma}{I}R\right]\frac{1}{E(k)}\sqrt{\pi a}$$

(A2) $$K_{B}=\left[F_{KB}\left(\frac{Mt}{2I}+\frac{Ma}{I}\right)-F_{LB}\frac{Ma}{I}\right]\frac{\sqrt{a/c}}{E(k)}\sqrt{\pi a}$$

(A3) $$R=\frac{k^{2}\sin\theta }{(1+k^{2})E\left(k\right)-{k^{\prime }}^{2}K\left(k\right)}=F(\theta ,a/c)$$

where *F_K_* is the SIF magnification factor for a semi-elliptical surface crack in a plate subjected to tension; *F_L_* is the SIF magnification factor for an elliptical crack in a semi-infinite solid subjected to linearly varying pressure on the crack surface; *E*(*k*) is the complete elliptic integral of the second kind; *K*(*k*) is the complete elliptic integral of the first kind; and *k* and *k*’ are the modulus and complimentary modulus of the Jacobian elliptic function, respectively, with *k*^2^ = 1 − *a*^2^/*c*^2^ and *k*’^2^ = 1 − *k*^2^.
